# Water deficit changes patterns of selection on floral signals and nectar rewards in the common morning glory

**DOI:** 10.1093/aobpla/plad061

**Published:** 2023-08-25

**Authors:** Yedra García, Benjamin S Dow, Amy L Parachnowitsch

**Affiliations:** Department of Biology, University of New Brunswick, 10 Bailey Dr, Fredericton, NB E3B 5A3, Canada; Department of Biology, University of New Brunswick, 10 Bailey Dr, Fredericton, NB E3B 5A3, Canada; Department of Biology, University of New Brunswick, 10 Bailey Dr, Fredericton, NB E3B 5A3, Canada

**Keywords:** Drought, *Ipomoea purpurea*, phenotypic selection, pollen limitation, resource limitation

## Abstract

Understanding whether and how resource limitation alters phenotypic selection on floral traits is key to predict the evolution of plant–pollinator interactions under climate change. Two important resources predicted to decline with our changing climate are pollinators and water in the form of increased droughts. Most work, however, has studied these selective agents separately and in the case of water deficit, studies are rare. Here, we use the common morning glory (*Ipomoea purpurea*) to investigate the effects of experimental reduction in pollinator access and water availability on floral signals and nectar rewards and their effects on phenotypic selection on these traits. We conducted a manipulative experiment in a common garden, where we grew plants in three treatments: (1) pollinator restriction, (2) water reduction and (3) unmanipulated control. Plants in pollinator restriction and control treatments were well-watered compared to water deficit. We found that in contrast to pollinator restriction, water deficit had strong effects altering floral signals and nectar rewards but also differed in the direction and strength of selection on these traits compared to control plants. Water deficit increased the opportunity for selection, and selection in this treatment favoured lower nectar volumes and larger floral sizes, which might further alter pollinator visitation. In addition, well-watered plants, both in control and pollinator deficit, showed similar patterns of selection to increase nectar volume suggesting non-pollinator-mediated selection on nectar. Our study shows that floral traits may evolve in response to reduction in water access faster than to declines in pollinators and reinforces that abiotic factors can be important agents of selection for floral traits. Although only few experimental selection studies have manipulated access to biotic and abiotic resources, our results suggest that this approach is key for understanding how pollination systems may evolve under climate change.

## Teaser

To compare the potential effects of two aspects of climate change, increased drought and reduced pollinators, we experimentally decreased pollinator access and water availability to flowers of the common morning glory. We measured both phenotypic changes and selection on floral signals and nectar rewards. Our findings show that water deficit not only altered flowers by reducing their size and nectar amount but also changed the natural selection patterns in these floral traits suggesting that these effects may have lasting evolutionary impacts. For our study, both phenotypic changes by water deficit and the subsequent natural selection were also much stronger than those imposed by pollinators' decline. We highlight the importance of considering both biotic and abiotic factors when addressing the ecological and evolutionary impacts of climate change on plant pollination.

## Introduction

The evolution of floral diversity is often hypothesized as the result of natural selection by pollinators (Faegri and Pijl 1979; Fenster *et al.* 2004). Indeed, patterns of directional selection are common in traits that act as signals to pollinators such as floral size and floral display (Parachnowitsch and Kessler 2010; Sletvold and Ågren 2010), and increasing evidence suggests similar patterns for other floral signals such as scent (Parachnowitsch *et al.* 2012; Opedal *et al.* 2022) and colour (e.g. increased brightness, Sletvold *et al.* 2016; Trunschke *et al.* 2021). A recent meta-analysis by Caruso *et al.* (2019) shows pollinator-mediated selection on floral traits is stronger than selection by other biotic agents such as herbivores. Pollinator-mediated selection also likely acts to shape floral nectar rewards, although there are still few estimates on this important trait (Parachnowitsch *et al.* 2019). Nectar traits are indeed integrated into the concept of pollination syndromes, where plants pollinated by similar functional groups of pollinators tend to converge on similar floral traits (Fenster *et al.* 2004).

The key role of pollinators in shaping floral evolution highlights the importance of investigating how their declines may modify patterns of selection on floral traits. This is particularly relevant in the current context of global change and anthropogenic disturbances that are expected to lead to pollinator declines in different ecosystems (Aizen *et al.* 2002; Rafferty 2017). In animal-pollinated flowers, decreased pollinator access may result in pollen limitation and lower seed production, leading to increased competition for pollinators that could alter selection on floral traits (Ashman and Morgan 2004; Sletvold and Ågren 2016; Caruso *et al.* 2019). In fact, if the intensity of selection on floral traits increases, plants may evolve in response to a decrease in pollinators (Thomann *et al.* 2013; Sletvold and Ågren 2016; Hossack and Caruso 2023). However, the magnitude of these effects on the intensity of selection may vary depending on whether trait variation is already reduced by past selection (Schluter 1988) and on the degree of pollen limitation affecting plant reproduction (Sletvold and Ågren 2016). Under modest pollen limitation, selection may act on traits that enhance pollinator attraction and pollen receipt (Caruso *et al.* 2005; Sletvold and Ågren 2016), while under strong pollen limitation, selection may promote traits that provide reproductive assurance through autonomous selfing (Fishman and Willis 2008; Panique and Caruso 2020). For instance, in an experiment with the orchid *Gymnadenia conopsea* under varying levels of pollen limitation, Sletvold and Ågren (2016) found that selection patterns on traits such as plant height, floral size and spur length were stronger with increasing pollen limitation. In *Impatiens capensis*, an experimental reduction in pollinators’ access led to stronger selection on traits that promote both outcrossing (larger chasmogamous flowers) and selfing (higher number of cleistogamous flowers, Panique and Caruso 2020). Limiting pollinator access in animal-pollinated flowers may also modify floral phenotypes. For instance, floral traits such as nectar content or floral colour may change after a pollinator visit and reduced pollination might induce phenotypic variation among visited and non-visited flowers (Ne’eman 1995; Veits *et al*. 2019; Santana *et al*. 2022). Given that anthropogenic disturbances may intensify pollen limitation in flowering plants, experimental studies exploring the effects of pollinators decline on floral traits and their evolution are needed (Bennett *et al.* 2020).

In addition to pollinators, the abiotic environment is an important factor shaping the evolution of floral traits (Strauss and Whittall 2006; Caruso *et al.* 2019) and can be as strong as selection driven by pollinators (Caruso *et al.* 2019). Limiting water availability can decrease plant–pollinator populations, cause phenological mismatch between plants and pollinators and modify floral traits that alter flower attractiveness (Carroll *et al.* 2001; Burkle and Runyon 2016; Gallagher and Campbell 2017; Descamps *et al.* 2021; Kuppler and Kotowska 2021). In a recent meta-analysis, Kuppler and Kotowska (2021) found consistent reductions in floral trait values under water stress for 8 of 10 morphological traits, as well as for nectar volume. These changes in the expression of floral traits may have further evolutionary implications by altering the distribution of floral phenotypes in the population where selection acts (Steele *et al.* 2011) and/or by decreasing the population mean fitness and thus increasing the opportunity for selection (Rundle and Vamosi 1996; Caruso *et al.* 2019). However, it remains unclear how such water stress-induced changes influence the strength of natural selection on floral traits as empirical tests are rare (e.g. Sherrard and Maherali 2006; Brachi *et al.* 2012; Lambrecht *et al.* 2017; Wu *et al.* 2023). The few studies measuring phenotypic selection on floral traits after experimental water manipulation have mainly focused on phenological traits and show contrasting results (e.g. Sherrard and Maherali 2006; Brachi *et al.* 2012). In *Arabidopsis thaliana*, selection intensity for earlier bolting and flowering increased with the severity of water stress (Brachi *et al.* 2012), while for *Avena barbata*, selection for early flowering was stronger in well-watered than drought plants (Sherrard and Maherali 2006). Given that climate change is increasing drought in different ecosystems, understanding the evolutionary consequences of water deficit on floral traits is urgent. We lack studies of water deficit on phenotypic selection on floral traits acting as signals (e.g. floral size, colour) and rewards (e.g. pollen, nectar) to pollinators, which is a crucial step to understand the consequences of climate change on pollination.

A powerful approach to explore functional links between floral phenotype and plant fitness is to combine phenotypic selection studies with experimental manipulation of the potential selective agent (Fishman and Willis 2008; Sletvold 2019). These studies explicitly test whether a potential agent of selection, biotic or abiotic, is influencing the observed relationship between plant fitness and the studied trait (Fishman and Willis 2008). Experimental approaches can reveal relationships between environmental factors and patterns of selection not detected in observational studies (Caruso *et al.* 2017). Manipulative studies comparing multiple agents of selection are needed to help understand the relative importance of factors that may have independent or combined effects on selection on floral traits (Caruso *et al.* 2005; Dai and Galloway 2013; Sletvold 2019). For instance, most of this work has focused on patterns of selection driven by pollinators and abiotic factors through experimental manipulation of pollen (e.g. pollen addition, reduced pollinator’s access) and nutrient resources (Caruso *et al.* 2005; Dai and Galloway 2013; Sletvold *et al.* 2017). However, to our knowledge, no previous studies have addressed patterns of selection on floral signals and nectar rewards driven by limitations in pollinator and water availability to test these predicted effects of climate change.

Here, we investigate the effects of reduction in pollinator access and water availability on floral traits including both signals (floral size, floral colour properties such as petal brightness) and rewards (nectar volume, nectar concentration) that mediate plant–pollinator interactions and their effects on phenotypic selection on these floral traits. Despite the important function of nectar in plant–pollinator interactions, information linking nectar rewards to plant fitness is still scarce and few studies have attempted to address natural selection acting on nectar traits (reviewed by Parachnowitsch *et al.* 2019). As study system, we used the common morning glory, *Ipomoea purpurea* (Convolvulaceae), an annual vine often used as a garden plant. Bumblebees are the main pollinators of *I. purpurea* and the species offers nectar as reward (Galetto and Bernardello 2004). Flowers of *I. purpurea* show a marked variation on floral traits, reinforcing the importance of phenotypic selection estimates to understanding the evolution and maintenance of its variation (Fry and Rausher 1997; Lau *et al.* 2008; Chaney and Baucom 2020). We addressed the following questions: (1) Does pollinator restriction and/or water deficit modify floral signals and rewards in *I. purpurea*? (2) What are the patterns of phenotypic selection on floral traits in *I. purpurea*? and (3) Do patterns of selection change under pollinator restriction or water deficit? We addressed these questions using seeds from different commercial sources planted in a common garden at the University of New Brunswick. We experimentally imposed two treatments, pollinator restriction and water reduction, by limiting plants’ access to pollinator visits and water. In addition, we established a control treatment with unmanipulated plants.

## Material and Methods

### Study system

The common morning glory *I. purpurea* (Convolvulaceae) is an annual vine native to Mexico and Central America. It is a weed in agricultural fields and disturbed areas across south-eastern USA (Smith and Rausher 2007). A popular garden plant outside of this range, it is commonly grown in New Brunswick. Flowering generally occurs in summer until the first frost. *Ipomoea purpurea* flower colour ranges from white to dark purple. Flowers are hermaphroditic, open early in the morning and senesce the same afternoon. The species is self-compatible with a mixed-mating system (Smith and Rausher 2007). Primary pollinators are bumblebees (*Bombus* spp., Rausher and Fry 1993).

### Experimental set-up

In spring 2021, we grew *I. purpurea* in a greenhouse at the Bailey Hall Biology building (University of New Brunswick). Seeds were sourced from six commercial suppliers from USA, Canada and UK because we did not have access to wild seed, and we wanted to increase our potential for genetic diversity; four were mixed-colour variety seeds and two were one variety **[see**Supporting Information—**Table S1****]**. We germinated 500 seeds, prioritizing sources with mixed colour. Seeds were scarified with sandpaper and sowed into 36-well trays with potting soil (VPW30, Greenworld). After 2 weeks, seedlings were transplanted (10.5 × 10.5 cm pots) and grown 4 weeks before moving to an experimental plot outside Bailey Hall. In July 2021, 400 plants were transplanted into individual 3.8 L fabric bags with ~5.2 g of slow release (Osmocote Classic 14-14-14) added. We provided plants bamboo stakes for support. All plants were watered as needed until the treatments began in mid-August.

On August 12th, we started treatments in a common garden on the east side of the Bailey Hall building. The plot was next to the building and managed lawns and landscaped garden beds that provide floral resources for pollinators, either intentionally (e.g. small wildflower garden next to the site) or unintentionally (e.g. weedy flowers in the lawns). Pollinators were common and throughout flowering at least two species of bumblebees, likely *Bombus impatiens* and *B. ternarius*, were frequently seen visiting flowers of the study plants. We arranged plants 30 cm apart with 1–1.5 m spacing between rows. Space limitations meant we had 12 rows of 15 plants and 36 rows of 5 plants. We applied treatments in a blocked design with eight blocks of 45 plants; two blocks had only unmanipulated plants and six blocks had 15 replicates of each experimental treatment: control, pollinator restriction and water deficit (*N* = 180 control, *N* = 90 pollinator restriction, *N* = 90 water deficit, Table 1). To control for potential variation in seed source, we matched these across treatments within blocks. We arranged the six blocks with three treatments in a stratified random design to ensure that each treatment had replicates from the different sources. Because of the low number of plants that flowered in one of the experimental blocks (*N* = 7 of 45 plants), we excluded this block from analyses (leaving *N* = 165 control plants, *N* = 75 pollinator restriction, *N* = 75 water deficit).

**Table 1.** *Ipomoea purpurea* corolla width and length measurements. Sample sizes per treatment at the start of the experiment (initial), plants with phenotypic measurements of floral size, nectar and stem diameter, petal reflectance measurements and fitness estimates (used in selection analyses).

	Treatment	Initial	Phenotypic measurements	Reflectance measurements	Selection estimates
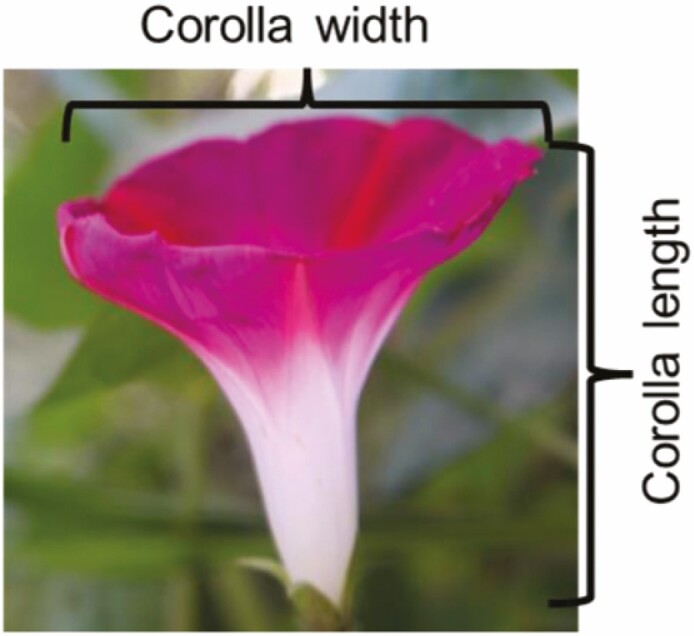	Control	180	162	121	155
	Water deficit	90	74	51	69
	Pollinator restriction	90	71	43	69

We watered control and pollinator restriction treatments every other day as needed, while the water deficit treatment dried down 12 days and thereafter was watered weekly, similar to Atala and Gianoli (2009). To reduce natural precipitation, we enclosed all fabric pots in tied plastic bags. We watered after trait measurements to reduce any immediate influence on traits. We used a soil moisture sensor (SM150T Delta-t Devices) inserted into the centre of each plot to measure soil moisture weekly beginning at the treatment set-up. We analysed variation in soil moisture among treatments with mixed ANOVA including treatment as predictor variable and date of moisture measurement as a random factor. To restrict pollinator access, we covered all buds with mesh bags.

### Phenotypic measurements

We measured floral traits based on block, starting with control plant blocks. For blocks with treatments, each sampling date, we measured plants from all treatments for 19 sampling dates. We measured corolla width (i.e. diameter) and corolla tube length to the nearest 0.01 mm with digital callipers for two fully opened flowers per plant (Table 1). To measure nectar, we covered floral buds with mesh bags the night before to exclude floral visitors. The following morning, we collected nectar from one to two flowers per plant (depending on how many opened) with 3 or 5 µL capillary tubes (Drummond Scientific) using the known volume to convert nectar length to volume (μL). We used a hand-held refractometer (Palm Abbe Misco) to measure nectar concentration (%). We averaged nectar values in plants with two nectar measurements. We also scored plants into seven floral colour categories distinct to human eye (white, blue, dark purple, violet, pale violet, pink and pale pink). As a proxy of plant size, we measured the diameter of the main stem at the end of flowering, as stem diameter had a strong positive correlation with plant biomass (wet weight) in a subset of 32 non-experimental plants (*r* = 0.86, *P* < 0.001).

After flowering, we counted the initiated and mature fruits per plant to estimate total fruit number. We counted seeds in ten mature fruits per plant and weighted the seeds in five of those fruits. For female fitness, we used seed set estimated as mean number of seeds per fruit × total fruits (initiated + mature).

#### Floral reflectance and bee vision

In addition to floral colour categories, we measured spectral reflectance in a subset of experimental plants (*N* = 215). One flower per plant was haphazardly selected and measured with a UV–Vis spectrophotometer coupled to a deuterium-halogen light source (Ocean Optics, USA). We measured floral reflectance (wavelength range 300–650 nm) by fixing the light source at a 45° angle to the petal. We took three measurements of the main colour for each petal, haphazardly chosen but excluding the nectar guides, and averaged these for a single measurement per plant (Palmqvist *et al.* 2021).

To assess variation in floral reflectance, we applied the visual sensitivity model based on Chittka (1992) to the mean reflectance spectra with the package ‘pavo 2’ in R (Maia *et al.* 2019). We only included plants from five colour categories (*N* = 213) due to small sample size of blue and white flowers. To test whether differences in reflectance were likely detected by bees, we applied the visual sensitivity of *B. terrestris* to estimate the chromatic contrast, which is related with colour conspicuousness in bees (Skorupski *et al.* 2007) as bumblebees of *Bombus* spp. are the main pollinators. We used the recommended settings for the bee colour hexagon based on a green background and daylight illumination (Maia *et al.* 2019). Bumblebees can chromatically distinguish two colours or a colour target from the background when the distance in their colour loci is >0.09 hexagon units (Dyer 2006). Chromatic contrast was measured as the Euclidean distance from a given stimulus to the achromatic centre in the colour hexagon (Chittka 1992) with the package ‘pavo’ in R (Maia *et al.* 2019).

#### Statistical analyses

First, we estimated Pearson’s correlation coefficients among the studied traits adjusted by Holm’s correction for multiple testing. Because the high positive correlation between corolla width and length (*r* = 0.78, *P* < 0.001), we used the geometric mean as a measure of floral size in the analyses. Stem diameter was log-transformed to improve normality.

Statistical analyses were performed in R 4.1.1 (R Development Core). In all linear (LMM) and generalized mixed models (GLMM, see below), we used the ‘lme4’ package in R (Bates *et al.* 2022) and tested them with type III Wald likelihood ratio test with the ‘car’ package (Fox *et al.* 2021). We performed Tukey post hoc pairwise comparisons with Holm correction with package ‘lsmeans’ (Lenth *et al.* 2019). Figures were done with the R package ‘ggplot2’ (Wickham *et al.* 2016) and edited with Inkscape v.1.1 (free open-source SVG graphics editor).

#### Effects of pollinator restriction and water deficit on floral traits and plant fitness

To test whether floral size, stem diameter, nectar volume and nectar concentration differed among treatments we ran separate LMMs that included each trait as response variable and treatment (i.e. control, pollinator restriction or water deficit) as an explanatory variable. Block, seed source and sampling date were included as random factors, with source nested within block. To address the effects of the experimental treatments on plant reproductive success, we run similar LMMs including total fruit production, mean seed weight and seed set as response variables, with block and source nested within block as random factors. Preliminary LMMs did not show significant effects of floral colour category nor of its interaction with experimental treatment on floral traits or plant reproductive measurements and thus were excluded.

To test for variation in petal spectral reflectance and determine the factors driving that variation, we performed a permutational multivariate analysis of variance (PERMANOVA,5000 permutations) on the Bray Curtis distances of the whole reflectance spectra as response variable and seed source, floral colour category and treatment as fixed factors with the package ‘vegan’ in R (Oksanen *et al.* 2017). We also included the interaction between colour category and treatment in the PERMANOVA. We considered the whole spectra between 300 and 650 nm and included each wavelength as a single data point (Palmqvist *et al.* 2021). We then ran a similar PERMANOVA using the Euclidean distance of the spectral reflectance data estimated with the visual model of *B. terrestris*. To determine whether pollinators could discriminate between colour categories, we first tested for variation in chromatic contrast between categories with a PERMANOVA on the pairwise chromatic distances (Maia and White 2018). We then followed a bootstrap approach on the mean chromatic distances between colour categories against the bee discrimination threshold (Maia and White 2018).

#### Phenotypic selection analyses

We estimated the opportunity for selection through female fitness in each treatment as the variance in relative fitness (individual seed set divided by mean seed set). To quantify phenotypic selection on the studied traits, we estimated linear selection gradients (β) by fitting multiple regression models (Lande and Arnold 1983). Relative fitness was included as response variable, and variance-standardized traits (mean 0 and variance 1) including stem diameter, floral size, nectar volume and concentration, as explanatory variables. We included block and source as covariates. Fitness was relativized and traits were standardized separately within each treatment. We ran additional selection models including floral colour category as a covariate and found similar patterns of selection **[see**Supporting Information—**Table S2****]**. We estimated non-linear selection by adding quadratic terms to the linear selection models for each treatment. To obtain non-linear selection gradients (γ), we doubled the non-linear partial regression coefficients (Stinchcombe *et al.* 2008). Finally, we quantified correlational selection by adding interaction terms to linear selection models. We restricted the correlational selection analyses to plants from the control treatment due to smaller sample sizes in the other treatments. To test whether direction and strength of selection varied among experimental treatments, we used ANCOVAs. Because our design was unbalanced, we ran separate ANCOVAs that compared each experimental treatment to the control.

Finally, to measure phenotypic selection on floral colour we used the set of control plants for which we had spectral data (*N* = 113). We used a dimensionality reduction approach on colour reflectance data following previous selection studies on floral colour in bee-pollinated species (Caruso *et al.* 2010; Renoult *et al.* 2013; Wassink and Caruso 2013; reviewed by Trunschke *et al.* 2021). To reduce the dimensionality of spectral data, we used a principal component analysis (PCA) on floral reflectance with the package ‘ade4’ in R (Dray and Dufour 2007). Previous work has shown that floral brightness is usually highly correlated with the first principal component PC1, while chromatic values are correlated to other PCs (Caruso *et al.* 2010; Renoult *et al.* 2013; Wassink and Caruso 2013). Thus, this approach allows to estimate selection on quantitative components of floral colour that can be perceived and selected by bee pollinators (reviewed by Trunschke *et al.* 2021). We focused on these colour properties rather on the chromatic contrast from the bee hexagon as we were interested in selection on colour traits that can be perceived by bees rather on selection on bee perception of these traits (Brunet *et al.* 2021). We chose the first three principal components that accounted for 89.91 % of variation in petal reflectance. We then fitted a multiple regression model that included the scores on these PCs from colour data together with floral size, nectar rewards and stem diameter.

To inspect multicollinearity in the selection models, we calculated variance inflation factors (VIFs) for the linear terms with the package ‘car’ in R (Fox *et al.* 2021). All VIFs were ≤ 2.5. We also conducted selection analyses by using mean-standardized traits instead of variance-standardized traits (Opedal 2021), detecting similar patterns of selection **[see**Supporting Information—**Table S3****]**.

## Results

### Pollinator restriction and water deficit effects on plant traits

**Figure 1.**
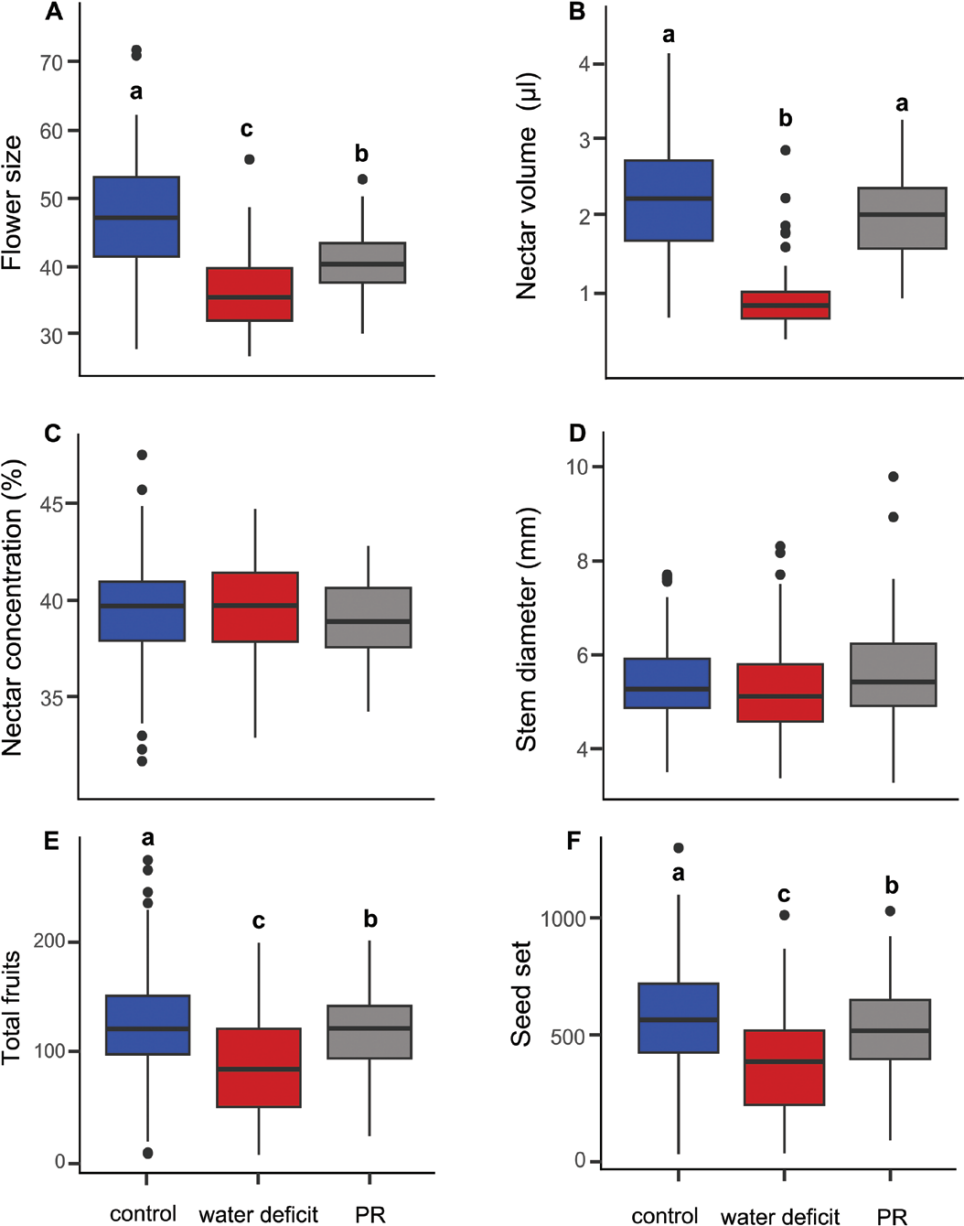
Variation in (A) flower size, (B) nectar volume, (C) nectar concentration, (D) stem diameter, (E) total fruit number and (F) seed set in *Ipomoea purpurea* plants from three experimental treatments. Different letters denote statistical differences in Tukey post hoc comparisons. PR = pollinator restriction.

Experimental reduction of pollinators and water altered floral signals of *I. purpurea*, while only water reduction altered floral rewards. Soil moisture in water deficit was 34.5 % lower compared to well-watered conditions (control vs water deficit: *T* = 78.02, *P* < 0.001; pollinator restriction vs water deficit: *T *= 65.20, *P* < 0.001) while well-watered plants from the control and pollinator restriction treatment did not show significant differences in moisture (control vs pollinator restriction, *T* = 1.53, *P* = 0.13). Flowers in water deficit were 23.5 % smaller than flowers from control plants (Fig. 1A, Supporting Information—**Table S4**) and also had a reduced nectar volume by ~53 % (Fig. 1B, Supporting Information—**Table S4**). Interestingly, plants with restricted pollinator access also had smaller flowers than control, but not to the same degree as water deficit (Fig. 1A, **Supporting Information—Table S4**) and nectar did not differ between pollinator restricted and control plants. We did not detect effects on nectar concentration nor plant size estimated from the diameter of the main stem for either treatment (Fig. 1C and D, **Supporting Information—Table S4**). Trait correlations showed low to moderate values (*r* ≤ 0.40), with the strongest correlation between floral size and nectar volume (*r *= 0.40, **Supporting Information—Table S5**).

**Figure 2.**
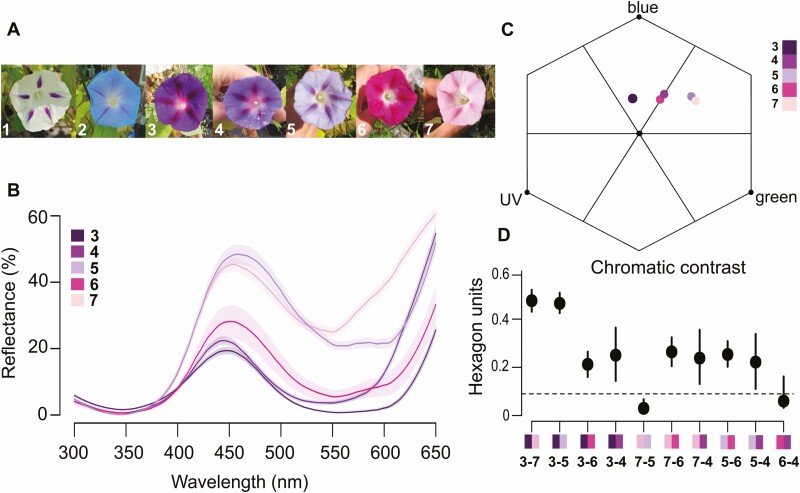
(A) Flowers of *Ipomoea purpurea* scored in seven colour categories (1–7: white, blue, dark purple, violet, pale violet, pink and pale pink). (B) Petal reflectance spectra for each of the five floral colour categories for which we had reflectance data (*N* = 213) with numbers match images in A. The shaded area denotes the standard deviation on reflectance (PERMANOVA: colour category *P *< 0.001). (C) Bee hexagon for *I. purpurea* flowers; centre represents the achromatic centre, edges show maximum excitation for ultraviolet (UV), blue and green photoreceptors. Coloured dots represent the mean of each floral colour category; colour scale with numbers match images in A. (D) Mean chromatic distance in hexagon units ± 95 % CI between each floral colour category pair (numbers match images in A). The dashed line represents the bee discrimination threshold (0.09 hexagon units), where points below the line can be interpreted as being perceived as the same in bee vision.

We detected significant differences in petal spectral reflectance among colour categories (**Supporting Information—Table S6**, Fig. 2B). We also found a significant discrimination in the chromatic contrast between categories (PERMANOVA: pseudo-*F*_4, 212_ = 72.56, *R*^2^ = 0.58, *P *= 0.001, Fig. 2C). Moreover, except for pink vs violet and their pale versions, differences in chromatic contrast between floral colour categories were likely detected by bumblebees as they were above the bee discrimination threshold (Fig. 2D). We did not find an interaction between floral colour category and treatment on spectral reflectance suggesting treatment did not alter reflectance within categories **[see****Supporting Information—Table S6****]**. The significant term for treatment in the model was likely due to different make up of colours across treatments **[see****Supporting Information—Tables S6 and S7****]**.

### Pollinator restriction and water deficit effects on reproductive success

Reduced pollinators and water access lowered plant fitness compared to control plants via fewer fruits and lower seed set (Fig. 1E and F, **Supporting Information—Table S4**). Our experiment was frequently visited by bumblebees, suggesting some outcrossing was likely for control and water deficit plants. However, abiotic conditions had a greater impact on reproductive success than eliminating pollinators; plants in water deficit had the strongest reduction in seed set (~33 %) compared to control plants (Fig. 1E). Pollinator restriction only reduced seed set by ~9 % compared to control plants suggesting that reproductive success in *I. purpurea* is more limited by abiotic resources than pollinators. Because all flowers in the pollinator restriction treatment were covered for their lifetime, these seeds are the result of selfing. For those seeds that were produced, the mean seed mass did not differ among treatments [**see****Supporting Information—Table S4**].

### Phenotypic selection

**Figure 3.**
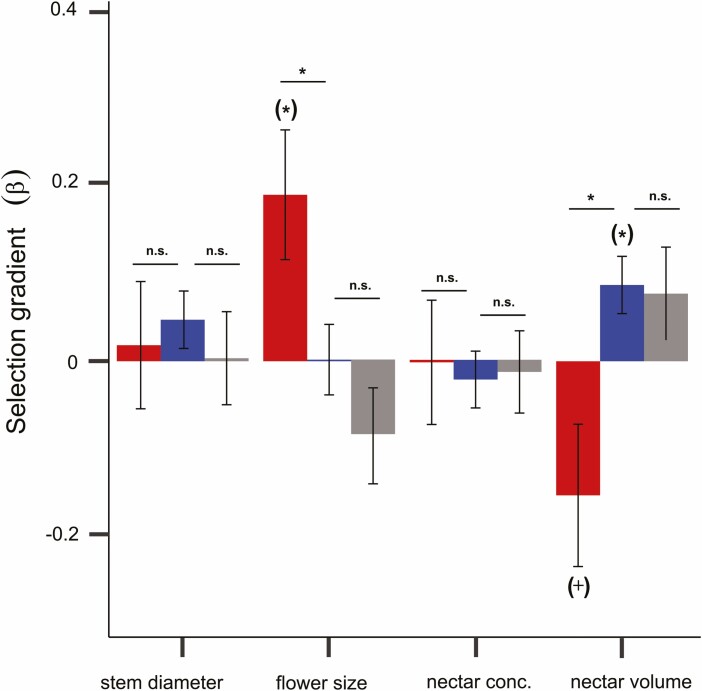
Phenotypic linear selection gradients of stem diameter, flower size, nectar concentration (nectar conc.) and nectar volume of *Ipomoea purpurea* plants in (i) water deficit (red), (ii) control (blue) and (iii) pollinator restriction (grey) treatments. Asterisks in parentheses (*) indicate the gradient is significantly different from zero (*P* < 0.05). ^+^*P* = 0.066. Symbols above lines show whether selection gradients are significantly different from each other when comparing the control treatment vs water deficit and control vs pollinator restriction (*P < 0.05, n.s. = nonsignificant, *P* > 0.05 from ANCOVA models).

Opportunity for selection via female fitness (variance of relative seed set) was higher for plants in water deficit (var = 0.34, SD = 0.58) than plants from the control (var = 0.15, SD = 0.40) and pollinator restriction treatments (var = 0.13, SD = 0.36). However, we did detect evidence of phenotypic selection for at least one trait in all treatments. The most striking contrast among treatments was selection on nectar volume. We found significant selection to increase nectar volumes in control plants, while in water deficit, selection for lower nectar volumes approached statistical significance (*P *= 0.066, Fig. 3, Table 2). Moreover, these selection gradients were significantly different (ANCOVA control vs water deficit: *F *= 5.43, *P *= 0.02, Fig. 3). Surprisingly because no pollinator visited or collected nectar from these plants, we detected similar trends of selection for increased nectar volume for plants with restricted pollinator access as control plants, but these were not significant (Fig. 3, Table 2).

**Table 2.** Linear (β ± SE) and non-linear (γ ± SE) selection gradients for four phenotypic traits of *Ipomoea purpurea* in three experimental treatments. ^+^*P* = 0.066, ^*^P < 0.05, ^**^P < 0.01.

Trait	Control		Water deficit		Pollinator restriction	
	β ± SE	γ ± SE	β ± SE	γ ± SE	β ± SE	γ ± SE
Floral size	0.003 ± 0.04	**−0.12 ± 0.06** ^*^	**0.19 ± 0.07** ^*^	0 ± 0.10	−0.08 ± 0.06	−0.04 ± 0.06
Nectar volume	**0.09 ± 0.03** ^**^	−0.01 ± 0.03	−0.15 ± 0.08^+^	0.02 ± 0.04	0.08 ± 0.05	−0.07 ± 0.04
Nectar concentration	−0.03 ± 0.03	0.005 ± 0.02	0 ± 0.07	0.04 ± 0.06	−0.02 ± 0.05	0.03 ± 0.04
Stem diameter	0.04 ± 0.03	−0.02 ± 0.04	0.006 ± 0.07	−0.10 ± 0.12	−0.03 ± 0.05	−0.16 ± 0.06^**^

Numbers in bold denote significant selection gradients

We observed selection for larger flowers in plants from the water deficit treatment where flowers were the smallest and this differed from the other two treatments (Fig. 3, Table 2, ANCOVA water deficit vs control: *F* = 9.08, *P *= 0.003, ANCOVA water deficit vs pollinator restriction: *F* = 9.10, *P *= 0.003). However, for control plants, floral size was under stabilizing selection although this pattern did not differ significantly among treatments (Table 2, ANCOVA control vs water deficit: *F *= 0.39, *P *= 0.54; ANCOVA control vs pollinator restriction: *F *= 0.80, *P *= 0.37), suggesting some costs to larger flowers in well-watered conditions. There was little selection detected in the diameter of the main stem despite its strong relationship to overall plant size. However, for plants in the pollinator restriction treatment, stem diameter was under stabilizing selection (Table 2). We did not find selection on nectar concentration in any treatment (Fig. 3, Table 2). Interestingly, for control plants where we had more power to detect patterns of correlational selection, we found selection for a negative correlation between floral size and nectar concentration (cor: −0.16 ± 0.08, *P *= 0.06). We found similar patterns of selection on floral traits when we added floral colour category to the models [**see****Supporting Information—Table S2**], and we did not detect selection on any of the three PCs of floral colour in control plants [**see****Supporting Information—Table S8**].

## Discussion

Our study shows that drought can not only change floral phenotypes but also has the potential to change floral evolution by altering the direction and strength of selection on floral traits. The most striking pattern of water deficit effecting both phenotypic traits and selection was seen for nectar rewards. As observed in other species, water deficit strongly reduced nectar volume in *I. purpurea* (Carroll *et al.* 2001; Gallagher and Campbell 2017; Suni *et al.* 2020; Höfer *et al.* 2021; Kuppler and Kotowska 2021). Because of carbon and water costs associated with nectar production, reducing nectar volume can help save resources especially in stressful dry environments (Pyke 1991; Wyatt *et al.* 1992). In fact, nectar volume can increase in response to experimental addition in abiotic resources such as water in *Asclepias syriaca* and nutrients in *Ipomopsis aggregata* (Wyatt *et al.* 1992; Burkle and Irwin 2009). Here, in addition to changes in the floral phenotype, we detected a fitness advantage to flowers with lower nectar volumes under water reduction. Hence, phenotypic selection to decrease nectar volume in *I. purpurea* in water deficit suggests an adaptive value of decreasing nectar rewards under limitation in abiotic resources. However, selection favouring less rewarding flowers might impact pollination if it leads to reduced pollinator visits (Gallagher and Campbell 2017; Höfer *et al.* 2021), suggesting there may be a trade-off between evolving in response to stressful environmental conditions and attracting pollinators (Strauss and Whittall 2006). Given that, to our knowledge, this is the first study to determine experimentally the effects of water deficit on selection on nectar rewards, further studies of selection on nectar in response to reduction in abiotic resources are necessary to assess the generality of our results.

In contrast to selection in water deficit, we found selection to increase nectar volume in *I. purpurea* from well-watered treatments, the control and pollinator restriction. Interestingly, selection to increase nectar volume even in the absence of pollinators suggests that selection on this trait was not mediated by pollinators in our study population. Despite pollinators having long been considered the primary drivers of nectar evolution in flowering plants, there is increasing evidence of relationships between nectar rewards and plant antagonists and microorganisms (Burdon *et al.* 2018; Vannette and Fukami 2018; Parachnowitsch *et al.* 2019). For instance, a previous study on *I. purpurea* has shown that the soil microbial community can change patterns of selection on flowering time and plant size, which opens the possibility to test whether soil microorganisms influence nectar traits in this system (Chaney and Baucom 2020). Overall, our findings add to few previous examples of selection on nectar rewards (Kulbaba and Worley 2012; Zhao *et al.* 2016; Parachnowitsch *et al.* 2019; Mackin *et al.* 2021). In sum, the little knowledge on nectar at the microevolutionary level, its role in mediating ecological interactions between plants and different organisms, and its plasticity to environmental variation call for further work on nectar responses in the context of climate change.

Unlike the marked effects on nectar volume, we found neither reduction in water nor in pollinators altered nectar concentration, similar to earlier studies on nectar rewards in drought conditions (Carroll *et al.* 2001; Gallagher and Campbell 2017; Rering *et al.* 2020). Maintaining nectar concentrations under changes in environmental factors may be beneficial as this trait is often associated with the frequency and length of pollinator visits (Carroll *et al.* 2001; Rering *et al.* 2020). Indeed, under experimental conditions bees can show stronger behavioural responses to variation in nectar concentration than in nectar volume suggesting that changes in nectar concentration may have a larger impact on pollinator visitation and thereby greater ecological costs (Cnaani *et al.* 2006). Different responses to water reduction for the nectar traits (volume vs concentration) could be because nectar volume but not concentration is positively correlated with floral size for *I. purpurea* (here, **Supporting Information—Table S5**; *r *= 0.41, *P* < 0.001, Galetto and Berbardello 2004), and thus, responses in nectar volume to water deficit are more likely to be constrained by responses in other floral traits. Interestingly, we also found marginally significant negative correlational selection between nectar concentration and flower size in control plants. Altogether, these varying patterns for nectar traits highlight the complexity of studying nectar rewards and thus, the need to consider different components of floral nectar when addressing variation in nectar and how it responds to changes in the environment.

Water deficit resulted in smaller flowers and selection favoured larger flowers, which was stronger and significantly different from control plants (Fig. 3 and Table 2). These plants in water deficit also had a higher opportunity for selection due to their reduced fitness (Arnold and Wade 1984; Caruso *et al.* 2017). Despite mounting evidence of plastic responses for floral traits to reduced water availability (Descamps *et al.* 2021; Kuppler and Kotowska 2021), whether these responses are adaptive has been rarely tested (Caruso *et al.* 2019). Our results differed from a greenhouse experiment with *Leptosiphon androsaceus*, where despite the lack of phenotypic plasticity in floral size, there was selection for smaller flowers in water reduction and larger flowers in well-watered conditions (Lambrecht *et al.* 2017). In our study, reducing water availability strongly decreased flower size in *I. purpurea* likely related to physiological stress and energetic costs of producing and maintaining large flowers in such conditions (Galen 2000; Teixido *et al.* 2016; Kuppler and Kotowska 2021). We also found stabilizing selection on floral size in plants from the control treatment suggesting a cost to large flowers (Parachnowitsch and Kessler 2010). If reducing water availability decreased mean floral size below the population optima, the selection to increase flower size could indicate a push back towards its optima. Pollinator-mediated selection on flower size can be common (Parachnowitsch and Kessler 2010; Sletvold and Ågren 2010; Caruso *et al.* 2019), but unfortunately, we cannot determine whether pollinators are selective agents on flower size in our study because of the lack of selection in control and pollinator-excluded plants. However, Bishop *et al.* (2023) found selection to increase floral size in northern populations of USA and increase mean values through time, suggesting *I. purpurea* flower size may be evolving in the wild.

Surprisingly, *I. purpurea* plants under restricted pollinator access also had smaller flowers than control plants, for which previous evidence is rare (but see Caruso *et al.* 2005). While we cannot exclude a bagging effect on floral development and size, we did not find differences in flower size from the pollinator restriction treatment sampled on different days (*F*_1, 69_ = 1.20, *P* = 0.28). Because bags were left covering the plants, later measurements had been bagged for longer, suggesting there may not be a strong developmental effect of the bags on flower size. Bagging also did not alter nectar, as has been observed in other species due to increasing temperature and humidity (Wyatt *et al.* 1992). While interesting, the selection patterns on floral traits without pollinators never differed from control plants with open pollination suggesting pollinators are not responsible for the patterns of selection observed in control plants.

We did not find evidence of phenotypic selection on floral colour in *I. purpurea*. Floral colour constitutes one of the most important visual signals mediating pollinator attraction in many pollination systems (Schiestl and Johnson 2013; Trunschke *et al.* 2021). Here, the low dependence on pollinators for female reproduction may explain the lack of phenotypic selection acting on continuous colour variation in our study. However, floral reflectance may be important in other contexts, for example, pale *I. purpurea* colour morphs attracted fewer pollinators and had lower outcrossing rates than dark morphs in a previous study (Brown and Clegg 1984). While the evolution of floral colour polymorphism in *I. purpurea* has been the focus of multiple studies (Fry and Rausher 1997; Mojonnier and Rausher 1997; Zufall and Rausher 2003), these are the first selection estimates on quantitative colour variation. Our results also emphasize the importance of measuring reflectance to understand differences in perceived colours because differences that seem important because we humans can see them may have little relevance to the insect visitors. Most studies measuring pollinator-mediated selection on continuous colour properties have not found significant selection (Trunschke *et al.* 2021). While the complexity of colour properties and flexible responses of pollinators to floral colour may explain the difficulty of detecting selection (Trunschke *et al.* 2021), other non-pollinator agents may drive selection on floral colour (Caruso *et al.* 2010; Sletvold *et al.* 2016). Here, we restricted our selection analysis on colour to control plants because of sample size limitations and uneven representation of the colour categories across treatments. However, drought can induce floral colour variation through changes in pigment concentration, and floral colour morphs can show different tolerance to water stress (Schemske and Bierzychudek 2001; Warren and Mackenzie 2001), suggesting understanding selection on colour in drought conditions could be an important goal for studying the effects of drought on flowers.

We found pollinator restriction had little effect on selection on floral traits, contrasting with the strong effects of water reduction. Earlier studies simulating pollinator declines have shown similar weak effects on selection on floral traits (Caruso *et al.* 2005, 2019; Sletvold and Ågren 2016; Hossack and Caruso 2023). In one of the few selection studies to also manipulate abiotic resources and pollinator access, Caruso *et al.* (2005) reported no differences in the strength of selection on floral traits in *A. syriaca* with reduced pollinator access, contrasting with changes in selection by nutrient addition. These and our results may be due to greater resource limitation than pollen limitation for seed production in these species (Caruso *et al.* 2005, 2017). Here, *I. purpurea* plants under restricted pollinator access set more fruits and seeds than plants in water reduction and only ~9 % fewer seeds than open-pollinated plants, indicating a much greater limitation by water than by pollinators. Hence, the opportunity for selection was similar in control and pollinator restriction treatments and greater in water deficit, where mean female fitness was the lowest compared to well-watered treatments (Fig. 1E and F). These findings add to increasing evidence showing that variation in selection is more likely the result of environmental factors with a large impact on mean fitness (reviewed by Caruso *et al.* 2017). However, our study seeds were garden varieties likely selected for high selfing rates (but see Mason *et al.* 2015 for similar results with seeds from natural populations), and selfing genotypes in *I. purpurea* have low levels of inbreeding depression (Chang and Rausher 1999). Thus, studies using seeds from natural populations or focused on species with stronger dependence on pollinators such as self-incompatible species, may provide a stronger test of the relative and interactive effects of pollinator and abiotic resource limitations on selection on floral traits (Sletvold 2019).

Our study shows that drought could affect the evolution of floral signals and nectar rewards, which may further impact patterns of pollinator visitation. While our results suggest drought may lead to faster evolutionary changes in plant pollination systems than pollinators decline, further experimental work on pollen-limited and/or self-incompatible species is needed. Our study reinforces using experimental approaches to uncover the relative role of different agents of selection acting on multiple floral traits. As climate change comprises both biotic and abiotic factors that may have interactive effects on selection on floral traits, we call for future studies considering multiple agents of selection in factorial-crossed experiments.

## Supporting Information

The following additional information is available in the online version of this article –

**Table S1.** Information on seed commercial suppliers of *Ipomoea purpurea*.

**Table S2.** Linear selection gradients on *Ipomoea purpurea* plants from three experimental treatments, with floral colour category included as blocking variable in the model.

**Table S3.** Mean-standardized linear and non-linear selection gradients on *I. purpurea* for four plant traits in three experimental treatments.

**Table S4.** Results of Wald-chi square test on the coefficients of LMMs and GLMMs testing for variation on floral traits between experimental treatments.

**Table S5.** Pearson’s correlations among four plant traits of *I. purpurea* from the unmanipulated control treatment (*N* = 162).

**Table S6.** PERMANOVA results for the spectral reflectance of *Ipomoea purpurea* petals before and after considering *Bombus terrestris* visual model, testing for the effects of commercial seed source, floral colour category, experimental treatment and the interaction of colour category and treatment.

**Table S7.** Sample size of each floral colour category in the experimental treatments.

**Table S8.** Linear selection gradients on a subset of *Ipomoea purpurea* plants from the control treatment (*N* = 113) with petal colour included in the multiple regression model.

plad061_suppl_Supplementary_Tables

## Data Availability

The data used in this article are available at Figshare: https://doi.org/10.6084/m9.figshare.23992404.v1

## Notes

**Natural History and Conservation. Chief Editor:** F. Xavier Picó
