# Supplementary material for: Water deficit changes patterns of selection on floral signals and nectar rewards in the common morning glory
Source: AoB Plants. 2023 Aug 25;15(5):plad061. doi: 10.1093/aobpla/plad061 (PMC10601024; doi:10.1093/aobpla/plad061)
Supplement: plad061_suppl_Supplementary_Tables [file plad061_suppl_supplementary_tables.pdf]

Appendix for: Water deficit changes patterns of selection on floral signals and nectar rewards in the common morning glory

Yedra García, Benjamin S. Dow & Amy L. Parachnowitsch

Table S1. Information on seed commercial suppliers of *Ipomoea purpurea*. Seeds denote number of seeds germinated from each source, final counts of plants in each treatment with phenotypic data/fitness data for selection estimates. Control plants received regular watering and open access to pollinators, WD = water deficit, PR = pollinator restriction.

Seed supplier	Country	*I. purpurea* variety	Seeds	Control	WD	PR
Seed Corner	USA (WA)	mixed	250	74/74	51/47	52/51
Refuge Mary Sue	Canada	Purple morning glory (dark purple)	20	4/4	6/5	5/5
Brother Nature	Canada (BC)	‘Grandpa Ott’ (dark purple)	70	32/30	11/11	8/8
Plant world Seeds	UK	mixed	30	2/-	1/1	1/-
Heritage Harvest Seed	Canada (MB)	mixed	100	29/28	5/5	5/5
Seeds and Spice	Canada	mixed	30	21/19	-	-

Table S2. Linear (β ± SE) selection gradients on *Ipomoea purpurea* plants from three experimental treatments, with floral colour category included as blocking variable in the multiple regression model. ***P* < 0.01, ****P* < 0.001.

**Trait**	**Control N= 155**	**Water deficit N=69**	**Pollinator restriction N=69**
Stem diameter	0.04 ± 0.03	0.09 ± 0.08	-0.03 ± 0.05
Floral size	0.003 ± 0.04	**0.32 ± 0.08***	-0.004 ± 0.05
Nectar concentration	-0.03 ± 0.03	0.03 ± 0.08	-0.02 ± 0.05
Nectar volume	**0.10 ± 0.03**	-0.14 ± 0.08	0.05 ± 0.05

Table S3. Mean-standardized linear (β ± SE) and non-linear (γ ± SE) selection gradients on *I. purpurea* for four plant traits in three experimental treatments. Control: N= 155, Water deficit: N= 69, Pollinator restriction: N= 69. *$P < 0.05$, **$P < 0.01$.

Trait	Control		Water deficit		Pollinator restriction	
	β ± SE	γ ± SE	β ± SE	γ ± SE	β ± SE	γ ± SE
Floral size	0.02 ± 0.25	**-2.47 ± 1.01***	**1.15 ± 0.45****	-0.001 ± 1.95	-0.72 ± 0.47	-1.09 ± 2.30
Nectar volume	**0.26 ± 0.10****	-0.12 ± 0.24	**-0.37 ± 0.17***	0.10 ± 0.17	0.50 ± 0.26	-0.80 ± 0.49
Nectar concentration	-0.45 ± 0.53	1.17 ± 4.76	-0.004 ± 1.09	9.57 ± 14.05	-0.38 ± 0.94	13.42 ± 14.93
Stem diameter	0.26 ± 0.20	-0.48 ± 0.89	0.03 ± 0.37	-1.21 ± 1.44	-0.17 ± 0.27	**-2.17 ± 0.70****

Table S4. Results of Wald-chi square test on the coefficients of LMMs and GLMMs testing for variation on floral traits between experimental treatments (control, water deficit, pollinator restriction). X^2, *P* values and degrees of freedom (in parentheses), are reported.

**Traits**	**Treatment**	
	**X^2 (2)**	***P***
Floral size	70.58	**<0.001**
Nectar volume	196.31	**<0.001**
Nectar concentration	2.50	0.29
Stem diameter	3.90	0.14
**Reproductive success**		
Total fruits	45.01	**<0.001**
Mean seed weight	0.51	0.77
Seed set	45.03	**<0.001**

Table S5. Pearson's correlations among four plant traits of *I. purpurea* from the unmanipulated control treatment (N= 162). Entries below the diagonal are *P* values adjusted for multiple comparisons. n.s: not significant.

	Stem diameter	Floral size	Nectar concentration	Nectar volume
Stem diameter		-0.02	0.03	0.02
Floral size	n.s		0.11	0.41
Nectar concentration	n.s	n.s		0.10
Nectar volume	n.s	<0.001	n.s	

Table S6. PERMANOVA results for the spectral reflectance of *Ipomoea purpurea* petals before and after considering *Bombus terrestris* visual model, testing for the effects of commercial seed source, floral colour category (i.e. colour cat.), experimental treatment and the interaction of colour category and treatment (colour cat. x treatment). DF: degrees of freedom.

	**Factor**	**DF**	**Sum of squares**	**pseudo-*F***	**R^2**	***P***
Spectral reflectance	source	5	2.66	9.87	0.11	**<0.001**
	colour cat.	4	9.71	45.06	0.40	**<0.001**
	treatment	2	0.64	5.90	0.03	**<0.001**
	colour cat. x treatment	8	0.49	1.14	0.02	0.31
	Residual	189	10.13		0.42	
Bombus model	source	5	2.01	10.57	0.11	**<0.001**
	colour cat.	4	8.46	55.39	0.45	**<0.001**
	treatment	2	0.66	8.64	0.03	**<0.001**
	colour cat. x treatment	8	0.32	0.02	1.03	0.41
	Residual	189	7.20		0.38	

Table S7. Sample size of each floral colour category in the experimental treatments.

**Colour category**	**Control**	**Water deficit**	**Pollinator restriction**
white	1	1	0
blue	1	0	0
dark purple	55	19	11
violet	1	4	4
pale violet	14	7	1
pink	36	10	13
pale pink	14	9	14

Table S8. Linear selection gradients (β ± SE) on a subset of *Ipomoea purpurea* plants from the control treatment (N= 113) with petal colour included in the multiple regression model as scores from the three principal components explaining 89.91% of variation on petal spectral reflectance. ***P* < 0.01.

**Trait**	**β ± SE**
Stem diameter	0.04 ± 0.04
Floral size	0.015 ± 0.04
Nectar concentration	-0.03 ± 0.04
Nectar volume	**0.09 ± 0.04****
PC1 colour	-0.03 ± 0.04
PC2 colour	-0.005 ± 0.04
PC3 colour	0.06 ± 0.04
